# Late-Onset Alzheimer’s Disease Genes and the Potentially Implicated Pathways

**DOI:** 10.1007/s40142-014-0034-x

**Published:** 2014-03-22

**Authors:** Samantha L. Rosenthal, M. Ilyas Kamboh

**Affiliations:** 1 Department of Human Genetics, Graduate School of Public Health, University of Pittsburgh, Pittsburgh, PA 15261 USA; 2 Alzheimer’s Disease Research Center, University of Pittsburgh, Pittsburgh, PA USA

**Keywords:** Late-onset Alzheimer’s disease, Genetics, Biological pathways

## Abstract

Late-onset Alzheimer’s disease (LOAD) is a devastating neurodegenerative disease with no effective treatment or cure. In addition to *APOE*, recent large genome-wide association studies have identified variation in over 20 loci that contribute to disease risk: *CR1, BIN1, INPP5D, MEF2C, TREM2, CD2AP, HLA*-*DRB1/HLA*-*DRB5, EPHA1, NME8, ZCWPW1, CLU, PTK2B, PICALM, SORL1, CELF1, MS4A4/MS4A6E, SLC24A4/RIN3,FERMT2, CD33, ABCA7, CASS4*. In addition, rare variants associated with LOAD have also been identified in *APP*, *TREM2* and *PLD3* genes. Previous research has identified inflammatory response, lipid metabolism and homeostasis, and endocytosis as the likely modes through which these gene products participate in Alzheimer’s disease. Despite the clustering of these genes across a few common pathways, many of their roles in disease pathogenesis have yet to be determined. In this review, we examine both general and postulated disease functions of these genes and consider a comprehensive view of their potential roles in LOAD risk.

## Introduction

Alzheimer’s disease (AD) is the sixth leading cause of death in the US. Healthcare costs in 2013 alone surpassed $200 billion (USD), and this figure is estimated to be over 1 trillion dollars by 2050 [www.alz.org]. AD is characterized by two pathological hallmarks in affected areas of the brain, extracellular deposition of senile plaques and intracellular occurrence of neurofibrillary tangles (NFTs), produced by abnormal aggregation of amyloid beta (Aβ) and hyperphosphorylation of tau, respectively. These plaques and tangles interfere with calcium signaling and synaptic transmission, induce a constant state of inflammation in the brain and ultimately lead to neuronal death. Patients with AD often initially present with mild cognitive impairment (MCI), which progresses to more severe memory loss and eventually loss of autonomy.

Alzheimer’s disease is a complex and multifactorial neurodegenerative disease and a leading cause of dementia among elderly people. However, a small number of individuals develop AD at a younger age, and because of this variation in age at onset, the disease is classified into early (<60 years age) and late (≥60 years age) onset forms. Early onset Alzheimer’s disease (EOAD) accounts for only 1–2 % of all AD cases, and it usually follows an autosomal dominant inheritance pattern where mutations in a single gene can cause the disease. To date, mutations in three genes, including amyloid precursor protein (*APP*), presenilin *(PS)*-*1* and *PS*-*2*, have been linked to EOAD [1–4].

Late-onset Alzheimer’s disease (LOAD) is much more common and far more complex than EOAD with the possible involvement of multiple genes and gene-gene and gene-environment interactions. Until 2009, *APOE* was the only established susceptibility marker for LOAD that accounts for ~25 of the estimated heritability of ~80 % [5]. This indicates the involvement of additional genetic factors that can modify the risk of LOAD. In order to identify the remaining genes for LOAD, efforts were focused on conducting genome-wide association studies (GWAS) because this approach is hypothesis free and conceptually would identify all known and unknown genes. However, with the exception of the *APOE* region, no other significant associations were replicated in earlier GWAS, indicating that the effect sizes of the remaining LOAD genes are small, and an exceptionally large number of cases and controls are required to identify additional genes. Since 2009, five large GWAS and a meta-analysis have identified significant associations of LOAD with SNPs in 20 additional loci, including *CLU*, *CR1,* *PICALM,* *BIN1,* *ABCA7, MS4A4, EPHA1, CD2AP CD33*, *INPP5D, MEF2C, HLA*-*DRB1/HLA*-*DRB5, NME8, ZCWPW1, PTK2B, SORL1, CELF1,SLC24A4/RIN3,FERMT2* and *CASS4* [6•, 7•, 8•, 9•, 10•, 11••].

Generally, these genes fall into at least one of three pathways–inflammatory, lipid metabolism and endocytosis–all of which have been suggested to play some role in disease. In addition to these genes where common variants are associated with disease risk, recent studies have identified rare variants in *APP* [12•], *triggering receptor expressed on myeloid cells 2* (*TREM2*) [13•, 14•] and *phospholipase D3* (*PLD3)* [15•] that also confer protection or risk against LOAD. Taken together, these findings produce a list of 24 genes/loci (Table 1), spread across the genome and loosely falling into the three common pathways, which mediate risk for LOAD. Below, we summarize the biology of each gene, postulate potential pathological mechanisms based upon their shared functions and regulatory networks, and suggest knowledge gaps that future studies may aim to fill.

**Table 1:** Late-onset Alzheimer’s disease (LOAD) genes

Chromosome position	Gene
1q32	*CR1*
2q14	*BIN1*
2q37.1	*INPP5D*
5q14.3	*MEF2C*
6p12	*CD2AP*
6p21.1	*TREM2* ^a^
6p21.3	*HLA*-*DRB5/HLA*-*DRB1*
7p14.1	*NME8*
7q22.1	*ZCWPW1*
7q34	*EPHA1*
8p21.1	*PTK2B*
8p21-p12	*CLU*
11p11	*CELF1*
11q12.1	*MS4A6A*
11q14	*PICALM*
11q23.2-q24.2	*SORL1*
14q22.1	*FERMT2*
14q32.12	*SLC24A4/RIN3*
19p13.3	*ABCA7*
19q13.2	*PLD3* ^a^
19q13.2	*APOE*
19q13.3	*CD33*
20q13.31	*CASS4*
21q21.3	*APP* ^a^

^a^Rare variants in these genes are associated with LOAD risk

### APOE (Apolipoprotein E)

The association between the *APOE* genotype and AD risk is the strongest and best replicated association for any AD risk locus where the APOE*4 is a risk allele and APOE*2 is a protective allele. Its association with LOAD risk was determined prior to and validated with genome-wide association studies. Some of the most attractive theories for the role of *APOE* in AD pathogenesis involve its roles in inflammatory response, oxidative stress and lipid levels [16]. Interestingly, research has shown that both the APOE*2 and APOE*4 alleles increase the risk of cerebral amyloid angiopathy (CAA) by encouraging accumulation of Aβ in the cerebral vasculature [17].

## Identification of LOAD Genes by Genome-wide Association Studies

In addition to the established association of *APOE*, recent GWAS have identified 20 genes/loci for LOAD. Below, we briefly discuss the known functions of the newly identified genes that might be relevant to AD biology. Since the GWAS approach generally identifies a genomic region associated with the disease and not necessarily the actual gene when there are multiple genes in a region, some of these genes should be considered provisional until confirmed by more in-depth functional tools.

### CR1 (Complement Component (3b/4b) Receptor 1)

CR1 is a major player in the immune system. It serves as a B cell receptor for fragments of complement components C3 and C4 and is involved in factor-I mediated cleavage of C3, and thus regulates complement activation [18, 19]. The exact relationship between CR1 function and LOAD is still unclear [20, 21]. Evidence for a role in brain vasculature as a means to mediate LOAD risk has been provided by Holton et al. [22], who were able to detect low *CR1* expression in white matter and cerebellum. Individuals with the genotype pattern *PICALM*-GG, *CR1*-GG, APOE*4 had decreased episodic memory, an endophenotype of LOAD, regardless of their affection status, providing further evidence of *CR1*’s role in LOAD risk [23].

### BIN1 (Bridging Integrator 1)

*BIN1* is a tumor suppressor gene that has been shown to be involved in a number of cancers, including hepatocellular carcinoma [24], melanoma [25], astrocytoma [26] and breast cancer [27]. Its known role in endocytosis [28] coupled with its association with LOAD makes it an attractive candidate gene for AD. In one study of sporadic AD, BIN1 protein levels were significantly lower in tissues from cases than from those of age-matched controls and neither overexpression nor knockdown of *BIN1* affected APP processing in SH-SY5Y cells [29]. This study, which found no effect of *BIN1* on tau pathology, differs from another by Chapuis et al. [30], which found BIN1 was increased in AD cases compared to controls and that the *Drosophila* ortholog to *BIN1*, *Amph*, mediates Tau-induced neurotoxicity. The role of *BIN1* in LOAD pathogenesis is still unclear and has been reviewed extensively by Tan et al. [31].

### INPP5D (Inositol Polyphosphate-5-Phosphatase, 145 kDa, aka SHIP1)

INPP5D plays a role in a number of inflammatory responses in addition to its regulation of cytokine signaling and inhibition of the PI3K-driven oncogenic pathway [32, 33]. Differential expression for this gene has been observed between classical Hodgkin’s lymphoma (cHL) cells and nodular lymphocyte predominant HL (nLPHL) cells, with the Hodgkin Reed-Sternberg cells of cHL showing decreased expression [34]. Furthermore, its expression has been shown to inhibit proliferation of acute myeloid leukemia cells [32]. Jickling et al. [35] have linked an increase in blood INPP5D with an increased risk of hemorrhagic transformation (HT) in ischemic stroke patients. HT is associated with increased blood-brain barrier permeability and thus may share some molecular mechanisms with AD. Of note is the binding of INPP5D with the product of another LOAD risk gene, *CD2AP,* in plasmacytoid dendritic cells (pDCs). This complex controls degradation of IgE receptor FcεRIγ [36]. As we discuss later in this review, some IgE receptors are members of the *MS4A* gene superfamily that has been associated with LOAD risk, indicating this complex and related pathways might be of importance to AD pathogenesis.

### MEF2C (Myocyte Enhancer Factor 2C)

Previously, *MEF2C* mutations have been correlated with distinct phenotypes, specifically that of del5q14 syndrome, which is similar to Rett syndrome and characterized by seizures, severe mental retardations and stereotypical movements [37, 38]. Sakai et al. [39] reported the first case of del5q14 syndrome in a Japanese adolescent male who exhibited a neuroendocrine phenotype and hypothalamic defects. Interestingly, the *Drosophila* ortholog of *MEF2C, Mef2*, has been shown to affect circadian behaviors and neuronal remodeling [40]. Since a number of AD patients experience disturbances in their circadian rhythms [41], it is possible that the association of *MEF2C* is reflective of this secondary phenotype of AD rather than pathogenesis itself.

### CD2AP (CD2-Associated Protein)

First identified as an adaptor protein with three Src-homology 3 (SH3) domains, *CD2AP* binds and clusters CD2 to facilitate junction formation between T cells and antigen-presenting cells [42]. Its role in endocytosis has been described, and its complex with cortactin links it to the cytoskeleton and vesicle movement [43]. This function makes *CD2AP* a prime candidate for modulating Aβ clearance. Increased susceptibility to neuritic plaque burden has been linked to *CD2AP* variation [44]. *CD2AP* behaves similarly to CIN85 (aka *SH3KBP1*) [43], the homologs of which have been identified as suppressors of Aβ toxicity in yeast and *C. elegans* [45]. Similarly, another study in *Drosophila* found that loss of the fly ortholog of *CD2AP* and *CIN85, cindr,* increased tau neurotoxicity in transgenic flies, further suggesting *CD2AP* normally functions in a protective role against AD risk [46].

### HLA-DRB1/HLA-DRB5 (Major Histocompatibility Complex, Class II, DR Beta 1/Major Histocompatibility Complex, Class II, DR Beta 5)

The *HLA*-*DRB1/HLA*-*DRB5* locus is a member of the major histocompatibility complex, a highly polymorphic region located on chromosome 6 that is responsible for numerous immune responses [47]. GWA studies have associated this locus with both multiple sclerosis [48] and another proteinopathy, Parkinson’s disease (PD) [49, 50]. Remarkably, knockout of MHCII in mice protects against α-synuclein-induced neurodegeneration, which is characteristic of PD [51]. While Parkinson’s and Alzheimer’s are two distinctly different diseases, both are characterized by neurodegeneration resulting from abnormal protein aggregation. Given the association of this locus with LOAD and the demonstration that MHCII signaling activates microglia in Parkinson’s [51], *HLA*-*DRB1/HLA*-*DRB5* may have a similar role in inflammatory responses that contribute to both pathologies.

### EPHA1 (EPH Receptor A1)

The EPHA1 receptor is a member of protein tyrosine receptors. There is high affinity for the receptor with its membrane-bound ligand, ephrin-A1. The interaction between EPHA receptors and ephrins is thought to play a role in synapse formation and development [52] and to regulate T cell interactions through integrin pathway [53]. Expression of *EPHA1* occurs in lymphocytes and epithelial cells and is downregulated in lipopolysaccaharide (LPS) fever-induced inflammation in rats [54]. A study of MCI individuals compared to healthy controls identified an association between Aβ deposition and *EPHA1* expression, with the C allele of rs11767557 being associated with decreased risk of being Aβ-positive. This association was only found in cognitively normal individuals, not those with MCI [55]. Overexpression of *EPHA1* also has been reported to produce more aggressive tumors in ovarian cancer [56].

### NME8 (NME/NM23 Family Member 8)

Defects in *NME8* (aka *TXNDC3*) have been associated with primary ciliary dyskinesia [57], and variation in this gene has been linked to increased bone mineral density and knee osteoarthritis risk [58, 59]. Work in mice has shown that deletion of the thioredoxin domains in sperm increases their age-related susceptibility to oxidative stress-induced phenotypes [60]. Literature on this gene is limited and indicates expression is primarily restricted to testis and respiratory epithelial cells [60, 61]. However, if expression of *NME8* was observed in the brain, the association between *NME8* and AD risk could be explained by variation that modifies its antioxidant action and subsequently alters the level of oxidative stress. Alternatively, variation in *NME8* could serve as an eQTL (expression quantitative trait loci) for other gene(s) whose expression is directly relevant to AD risk.

### ZCWPW1 (Zinc Finger, CW Type with PWWP Domain 1)

To date, only one paper has been published on *ZCWPW1*. He et al. [62] used solution NMR spectroscopy in the first and only determination of the 3D structure of ZCWPW1. This protein contains zf-CW domain, which has been identified in a number of other proteins responsible for chromatin remodeling and methylation states. The PWWP domain is also present in ZCWPW1 and, similar to the zf-CW domain, has been described in epigenetic regulations, indicating *ZCWPW1* as a histone modification reader [62]. Recently, a variant in *ZCWPW1* (rs1476679) associated with LOAD risk was also found to have functional relevance (RegulomeDB score: 1f), as it serves as an eQTL for *GATS, PILRB* and *TRIM4* and affects binding of CTCF and RFX3 [Rosenthal, unpublished data].

### CLU (Clusterin)

CLU, also referred to as apolipoprotein J (APOJ*)*, has been implicated in the formation of complexes that can cross the blood-brain barrier [63]. It is one of the primary chaperones for removal of Aβ from the brain [64]. AD patients have increased levels of CLU in the cortex and hippocampus [65, 66], so a link between increased levels of CLU and AD risk [67] is both expected and observed. Related to this, Thambisetty et al. [68] found an association between increased plasma clusterin and hippocampal atrophy, as well as disease severity and progression, suggesting its potential utility as a biomarker. In contrast, other studies show that the minor allele of the associated SNP, *CLU*/rs11136000, is associated with increased *CLU* expression but decreased AD risk, further complicating the relationship between CLU and Alzheimer’s disease [6•, 7•, 69, 70]. One possibility proposed by Ling et al. [69] is that for the increase in *CLU* expression to reduce risk, it must occur over the lifespan, prior to disease onset. In addition to its role in neurodegeneration, *CLU* expression affects chemotherapy resistance and severity of some cancers, which may be indicative of an inflammatory mechanism of action in AD, in addition to lipid trafficking [71–74]. Clusterin’s role in AD pathogenesis has been reviewed in depth by Nuutinen [64].

### PTK2B (Protein Tyrosine Kinase 2 Beta)

The *PTK2B* gene is located on chromosome 8 near the *CLU* gene. We originally reported that *PTK2B* was a potential new risk gene for AD, as we found multiple significant signals (albeit not genome-wide significant) in the *PTK2B* gene [75]. This observation has now been confirmed by the meta-analysis where *PTK2B* has been identified as a genome-wide significant locus for LOAD [11••]. PTK2B, also called PYK2, is a member of the focal adhesion kinase (FAK) family, a non-receptor protein tyrosine kinase family [76]. It responds to a number of stimuli and is subsequently activated by these stimuli through a combination of autophosphorylation and phosphorylation by Src-family kinases [77]. One such stimulus for *PTK2B* activation is changes in intracellular calcium levels, which are disrupted in AD brains [76, 78]. *PTK2B* indirectly regulates N-methyl-D-aspartate receptor (NMDAR) activity through src kinases [79, 80]. Work in mice suggests that loss of protein tyrosine phosphatase alpha (PTP-α), a regulator of *PTK2B*, can cause defects in NMDAR processes, including memory [81].

### MS4A4A/MS4A6E (Membrane-Spanning 4-Domains, Subfamily A, Member 4A/Membrane-Spanning 4-Domains, Subfamily A, Member 6E)

Perhaps the most interesting AD risk locus is the *MS4A* region located on chromosome 11. Despite its continued replication in multiple GWAS and original characterization over a decade ago, little else has been determined about this gene family [82, 83]. Members of the *MS4A* family of proteins have four transmembrane domains and are diversely expressed [82, 83]. Similarities between the structure and expression of mouse and human *HTm4* (*MS4A3*), another member of the *MS4A* family, have been demonstrated. Of interest is the expression of *HTm4* in the developing central nervous system of mice [84]. Expression of *MS4A6A* has been shown to correlate with Braak tangle and Braak plaque scores in AD patients, as was the minor allele *MS4A6E*/rs670139 [85]. Allen et al. [70] have identified variations in proxies of the genome-wide significant SNP rs670139 that increase *MS4A4A*’s expression in the brain and subsequently increase disease risk. Given the consistent replication of these loci with AD risk, it is essential that this gene family be studied with more functional techniques to assess its role in both normal and disease states.

### PICALM (Phosphatidylinositol Binding Clathrin Assembly Protein)

APP trafficking as well as Aβ clearance, specifically via clathrin-mediated endocytosis (CME), is one of the proposed pathways for affecting LOAD risk. Work in yeast and *C. elegans* has shown homologs of *PICALM* to be suppressors of Aβ toxicity [45]. More recently, Ando et al. [86] identified a link between *PICALM* and characteristic tau pathology of AD brains, specifically co-localization of PICALM with tau in NFTs, but not with pre-tangles or extracellular ghost tangles. Alternative splicing of *PICALM* yields three isoforms, and post-mortem studies of brain samples revealed a decrease in the levels of full-length PICALM and an increase in the shorter species in cases, indicating abnormal proteolysis of PICALM may affect Aβ clearance although no interaction between PICALM and Aβ was observed.

### SORL1 [Sortilin-Related Receptor, L(DLR class) A Repeats Containing]

Nearly a decade ago, Scherzer et al. [87] identified a link between decreased *SORL1 (LR11)* expression and AD. Microarray analysis of lymphoblast DNA showed a clear downregulation of *SORL1* expression in AD patients, and immunohistochemistry of AD brains exhibited decreased staining of pyramidal neurons and lowered protein levels in the frontal cortex. Additionally, single-site and haplotype association of *SORL1* with risk of amnestic mild cognitive impairment (aMCI), a common precursor to AD, has been reported in the Han Chinese [88]. Glerup et al. [89] have demonstrated SORL1 as an endocytic modulator of glial-derived neurotrophic factor (GDNF) and its related receptor, GFRα1.

### CELF1 (CUGBP, Elav-like Family Member 1)

CELF1 is largely implicated in myotonic dystrophy type I (DMI) because of its interaction with DMPK [90, 91]. However, Kim et al. [92] have shown that elimination of *CELF1* in transgenic mice with induced RNA toxicity does not completely alleviate features of DMI. *CELF1* has also been associated with certain types of cancer. For example, Talwar et al. [93] have observed overexpression of *CELF1* in oral squamous cancer cells results in a reduction of proapoptotic mRNA transcripts that ultimately leads to cell proliferation. The fly homolog of *CELF1, aret*, has been shown to mediate tau toxicity [46]. Recently, we have identified eight variants in linkage disequilibrium with the reported *CELF1*/rs10838725 that have suggestive functional relevance (RegulomeDB score: 1f) and are eQTLs for *C1QTNF4*. These data suggest that *CELF1* may be acting in conjunction with or serving as a proxy for other genes in this region that mediate AD risk [Rosenthal, unpublished data].

### SLC24A4/RIN3 (Solute Carrier Family 24 (Sodium/Potassium/Calcium Exchanger), Member 4/Ras and Rab Interactor 3)

SLC24A4 is a solute carrier that has been associated with pigmentation traits in European populations [94, 95]. Since *SLC24A4* is involved in iris development, it may also be involved in neuronal development and thus contribute to AD risk [96]. Two variants in this gene have been identified in Pakistani families with amelogenesis imperfecta (AI), and *Slc24a4* knockout mice have severe enamel defects, indicating a role for this solute carrier in amelogenesis [97]. Perhaps of most relevance to AD is the association of this gene with blood pressure in African Americans as AD may be influenced by vascular disease [98].

### FERMT2 (Fermitin Family Member 2)

FERMT2 (aka *kindlin*-*2*, *KIND2*) is a member of the Fermitin family of proteins, which are involved in cell–matrix adhesion complexes. FERMT2 can stimulate genomic instability, which ultimately facilitates breast cancer progression, and it also has been identified as a binding partner for KIND1, mutations in which are responsible for Kindler syndrome [99, 100]. Shulman et al. [46] independently validated the recent association of *FERMT2* with LOAD risk after performing a gene screen and in vivo studies in *Drosophila melanogaster*. Their work in flies shows altered expression of both *FERMT2* and *CELF1* homologs modulates Tau neurotoxicity as measured by a retinal phenotype and suggests biological relevance for these associations.

### ABCA7 [ATP-Binding Cassette, Sub-family A (ABC1), Member 7]

ABCA7 is a member of the ATP-binding cassette genes that are responsible for lipid transport, a particularly important function in the central nervous system [101]. Kim et al. [102–104] performed a number of mouse studies concerning the expression and function of *ABCA7*. Loss of *ABCA7* is not embryonic lethal and does not produce any clear irregularities in young mice, which is consistent with the late age at onset of AD. Their work has demonstrated that knockout of *ABCA7* does not affect cholesterol efflux by macrophages, nor is it sufficient to compensate when function of homologous lipid transporter, *ABCA1,* is lost. *ABCA7* expression is highest in the hippocampus, one of the earliest affected regions in the brains of AD patients, and microglia, the cells responsible for cerebral inflammatory response [102, 103]. ABCA7 also participates in macrophage uptake of Aβ, and ablation of *ABCA7* results in increased levels of insoluble Aβ [104]. ABCA7 also has been shown to mediate APP processing [105]. It remains to be seen whether the action of ABCA7 in AD is through its interaction with APOE and lipid metabolism, its function as an immune system molecule or a combination of both. *ABCA7* also has been associated with age at onset of AD [85]. A GWAS in African Americans found an effect size similar to *APOE* for *ABCA7*/rs115550680 (OR 2.31, *p* = 5.5 × 10^−47^, OR 1.79, *p* = 2.21 × 10^−9^, respectively), highlighting the diversity of genetic effects on different genetic backgrounds [106].

### CD33 (CD33 Molecule)

CD33 belongs to a class of immune cell surface receptors called sialic acid-binding immunoglobulin-like lectins (Siglecs). CD33 triggers immune cell–cell interactions through its own clathrin-independent endocytosis [107]. It has been shown that *CD33* expression is increased in AD brains [85], as is the number of CD33-positive microglia [108]. Both affected and asymptomatic carriers of the ‘C’ risk allele for the associated *CD33*/rs3865444 variant have a higher probability of being positive for Pittsburgh Compound B (PiB). Notably, carriers of ‘C’ risk allele also have a higher likelihood of obtaining an AD diagnosis, but the effects of the risk allele appear to have no bearing on tangle formation and are thus limited to plaque pathology, likely due to its inhibition of Aβ42 uptake and clearance by microglia [108, 109].

### CASS4 (Cas Scaffolding Protein Family Member 4)

CASS4 is a relatively understudied gene as evidenced by a mere three publications returned in a PubMed search for the gene and its alias, *HEPL*. The reported GWAS significant SNP, *CASS4*/rs7274581, is protective against AD, and a SNP in LD with this GWAS SNP, *CASS4*/rs6024870, shows evidence of regulatory function as well [Rosenthal, unpublished data]. CASS4 is a member of the CAS protein family, scaffolding proteins responsible for a number of cellular activities [110]. First characterized in 2008, CASS4 shares up to 42 % similarity in gene sequence with the other three members of the CASS family, but lacks the conserved YDYVHL motif. It is most highly expressed in spleen and lung tissues, as well as ovarian and leukemia cell lines, and its importance appears to be cell-specific and dependent upon the presence or absence other CAS family members’ expression [111]. *Dcas,* the *Drosophila* homolog to CAS family proteins, interacts with integrin pathway genes during early embryogenesis [112], and Kirsch et al. [113] have also demonstrated an interaction between another CAS family member, *CASS1*, and an established AD locus, *CD2AP*.

## Identification of LOAD Genes by Genomic Sequencing

In addition to *APOE*, GWAS have made significant contribution in identifying 20 genes/loci for LOAD, but together common variants in these 21 genes explain about half of the estimated ~80 % heritability of AD [5]. This finding is consistent with published data for different diseases and traits where GWAS explain only a small fraction of the estimated genetic variance [114, 115], partly because GWAS arrays are designed to capture mainly the common variants with low penetrance and not the rare variants having higher individual penetrance. In the post-GWAS era, it is becoming necessary to perform genomic sequencing (targeted sequencing, whole exome-sequencing or whole-genome sequencing) in order to detect functional rare variants not only in known genes, but this approach will also help to identify new genes for LOAD. Recently, the application of whole-exome sequencing has resulted in the identification of rare functional variants in three additional genes for LOAD, including *APP*, *TREM2* and *PLD3*.

### APP (Amyloid Precursor Protein)

The association between *APP* and AD is well established for EOAD; however, it was not until recently that a link between *APP* and the common LOAD was reported. APP is sequentially cleaved by α-secretase and then γ-secretase to produce amyloid intracellular domain and C3 fragments. Cleavage of APP by β-secretase rather than α-secretase produces a longer version of the peptide that is prone to aggregation and results in the formation of Aβ plaques characteristic of AD. A rare protective variant, A673T, has been identified in Icelandic and Finnish individuals with a strong effect size [12, 116]. Thus far, it seems the link between this rare variant is restricted to members of these populations as other studies have not detected this variant in other populations [117, 118]. However, these findings do suggest that *APP* may warrant a second look via sequencing to determine whether other rare variants in this gene exist that may explain some of the missing heritability for LOAD.

### TREM2 (Triggering Receptor Expressed on Myeloid Cells 2)

*TREM2* has only recently been added to the list of associated LOAD genes and stands out among the 24 identified loci because of a missense mutation that has a similar effect size to the APOE*E4 allele. The rare R47H variant was found in both the Icelandic [14•] population and an international cohort of European descent [13•]. A meta-analysis of these studies and others reports an odds ratio of 3.4 [119], further strengthening the case for *TREM2* as a major LOAD risk locus. Previously, mutations in *TREM2* have been associated with Nasu-Hakola disease (aka polycystic lipomembranous osteodysplasia with sclerosing leukoencephalopathy, PLOSL) [120, 121]. This rare recessive disease counts progressive frontal-type dementia among its clinical features, which makes the association of *TREM2* with LOAD interesting despite variations in age at onset and type of dementia [122]. The TREM2 receptor is expressed by microglia, and expression of its ligands, TREM2-L, is amplified in apoptotic neurons. Furthermore, inhibition of TREM2 activity decreases phagocytosis of these cells by approximately one third, suggesting the interaction between TREM2 and its ligands facilitates clearance of apoptotic neurons [123].

### PLD3 (Phospholipase D Family, Member 3)

The latest addition to the list of AD risk loci is *PLD3*. Its role in LOAD risk was first identified in a small study of 14 families with the variant *PLD3*/rs145999145 and was validated in a population-based study as increasing LOAD risk (OR = 2.10). This variant was also associated with age at onset [15•]. PLD3 is a signaling enzyme about which little has been described. Zhang et al. [124] have shown Akt phosphorylation is inhibited in myoblasts when *PLD3* is overexpressed, and Osisami et al. [125] have suggested its role in myogenesis may be specifically related to myotube formation. Remarkably, another risk locus, *PTK2B,* has been shown to participate in a signaling pathway responsible for Akt activation [126]. *PLD3* also has been identified as a potential modifier of *BRCA1* and *BRCA2* [127].

## Pathway Analysis

Using Ingenuity Pathways Analysis software (version 18030641, Ingenuity Systems, Inc., 2014), we were able to assess the wide variety of shared functions of these loci. We examined a total of 27 molecules representing the GWAS loci (Table 2). Not surprisingly, the most significant of these function or disease annotations were LOAD (*p* = 2.88E − 21) and AD (*p* = 2.05E − 15) with 9 and 14 molecules implicated, respectively (Table 3). After removing annotations with three or fewer molecules, we were left with a total of 36 nominally significant (*p* < 0.05) groups of genes across a number of categories and function annotations. Late-onset Alzheimer’s disease and Alzheimer’s disease remained the most significant, followed by engulfment of cells and leukocytes (*p* = 1.17E – 06, *p* = 1.68E − 06, respectively). Both of these annotations cite *APOE, INPP5D, TREM2, ABCA7* and *BINI* as molecules involved in these processes, with *CR1* and *PICALM* included in the broader “engulfment of cells” annotation. The disease annotation with the most molecules involved is cancer, with 18 of the 27 genes involved (*p* = 3.63E−03). Indeed, of the 36 groups of genes, 14 are related to cancer, immunity/immunological disease or inflammatory responses/inflammatory disease (Table 3).

**Table 2:** LOAD risk loci examined in IPA analysis

Symbol	Entrez gene name	Location
*ABCA7*	ATP-binding cassette, sub-family A (ABC1), member 7	Plasma membrane
*APOE*	Apolipoprotein E	Extracellular space
*BIN1*	Bridging integrator 1	Nucleus
*CASS4*	Cas scaffolding protein family member 4	Other
*CD2AP*	CD2-associated protein	Cytoplasm
*CD33*	CD33 molecule	Plasma membrane
*CELF1*	CUGBP, Elav-like family member 1	Nucleus
*CLU*	Clusterin	Cytoplasm
*CR1*	Complement component (3b/4b) receptor 1 (Knops blood group)	Plasma membrane
*EPHA1*	EPH receptor A1	Plasma membrane
*FERMT2*	Fermitin family member 2	Cytoplasm
*HLA*-*DRB1*	Major histocompatibility complex, class II, DR beta 1	Plasma membrane
*HLA*-*DRB5*	Major histocompatibility complex, class II, DR beta 5	Plasma membrane
*INPP5D*	Inositol polyphosphate-5-phosphatase, 145 kDa	Cytoplasm
*MEF2C*	Myocyte enhancer factor 2C	Nucleus
*MS4A4A*	Membrane-spanning 4-domains, subfamily A, member 4A	Other
*MS4A4E*	Membrane-spanning 4-domains, subfamily A, member 4E	Other
*MS4A6A*	Membrane-spanning 4-domains, subfamily A, member 6A	Other
*MS4A6E*	Membrane-spanning 4-domains, subfamily A, member 6E	Other
*NME8*	NME/NM23 family member 8	Cytoplasm
*PICALM*	Phosphatidylinositol binding clathrin assembly protein	Cytoplasm
*PTK2B*	Protein tyrosine kinase 2 beta	Cytoplasm
*RIN3*	Ras and Rab interactor 3	Cytoplasm
*SLC24A4*	Solute carrier family 24 (sodium/potassium/calcium exchanger), member 4	Plasma membrane
*SORL1*	Sortilin-related receptor, L(DLR class) A repeats containing	Cytoplasm
*TREM2*	Triggering receptor expressed on myeloid cells 2	Plasma membrane
*ZCWPW1*	Zinc finger, CW type with PWWP domain 1	Other

**Table 3:** IPA functional analysis of LOAD risk loci, gene groups > 4

Category	Functions	Diseases or functions annotation	*p* value	Molecules
Metabolic disease	Late-onset Alzheimer’s disease	Late-onset Alzheimer’s disease	2.88E−21	APOE, BIN1, CD2AP, CD33, CLU, CR1, EPHA1, MS4A4A, PICALM
Neurological disease	Late-onset Alzheimer’s disease	Late-onset Alzheimer’s disease	2.88E−21	APOE, BIN1, CD2AP, CD33, CLU, CR1, EPHA1, MS4A4A, PICALM
Psychological disorders	Late-onset Alzheimer’s disease	Late-onset Alzheimer’s disease	2.88E−21	APOE, BIN1, CD2AP, CD33, CLU, CR1, EPHA1, MS4A4A, PICALM
Metabolic disease	Alzheimer’s disease	Alzheimer’s disease	2.05E−15	ABCA7, APOE, BIN1, CD2AP, CD33, CLU, CR1, EPHA1, MS4A4A, MS4A4E, MS4A6A, MS4A6E, PICALM, SORL1
Neurological disease	Alzheimer’s disease	Alzheimer’s disease	2.05E−15	ABCA7, APOE, BIN1, CD2AP, CD33, CLU, CR1, EPHA1, MS4A4A, MS4A4E, MS4A6A, MS4A6E, PICALM, SORL1
Psychological disorders	Alzheimer’s disease	Alzheimer’s disease	2.05E−15	ABCA7, APOE, BIN1, CD2AP, CD33, CLU, CR1, EPHA1, MS4A4A, MS4A4E, MS4A6A, MS4A6E, PICALM, SORL1
Cellular function and maintenance	Engulfment	Engulfment of cells	1.17E−06	ABCA7, APOE, BIN1, CR1, INPP5D, PICALM, TREM2
Cellular function and maintenance	Engulfment	Engulfment of leukocytes	1.68E−06	ABCA7, APOE, BIN1, INPP5D, TREM2
Connective tissue disorders	Rheumatoid arthritis	Rheumatoid arthritis	4.54E−06	APOE, CD33, CLU, CR1, HLA-DRB1, MS4A6A, PTK2B, SORL1
Immunological disease	Rheumatoid arthritis	Rheumatoid arthritis	4.54E−06	APOE, CD33, CLU, CR1, HLA-DRB1, MS4A6A, PTK2B, SORL1^a^
Inflammatory disease	Rheumatoid arthritis	Rheumatoid arthritis	4.54E−06	APOE, CD33, CLU, CR1, HLA-DRB1, MS4A6A, PTK2B, SORL1^a^
Skeletal and muscular disorders	Rheumatoid arthritis	Rheumatoid arthritis	4.54E−06	APOE, CD33, CLU, CR1, HLA-DRB1, MS4A6A, PTK2B, SORL1
Cellular function and maintenance	Receptor-mediated endocytosis	Receptor-mediated endocytosis	9.40E−06	APOE, CD2AP, PICALM, SORL1
Cellular function and maintenance	Phagocytosis	Phagocytosis of antigen presenting cells	9.68E−06	ABCA7, BIN1, INPP5D, TREM2
Cell-to-cell signaling and interaction	Phagocytosis	Phagocytosis of antigen presenting cells	9.68E−06	ABCA7, BIN1, INPP5D, TREM2
Inflammatory response	Phagocytosis	Phagocytosis of antigen presenting cells	9.68E−06	ABCA7, BIN1, INPP5D, TREM2^a^
Cellular development	Proliferation	Proliferation of lymphocytes	1.16E−05	APOE, CD2AP, CD33, CR1, HLA-DRB1, INPP5D, MEF2C, PTK2B
Cellular growth and proliferation	Proliferation	Proliferation of lymphocytes	1.16E−05	APOE, CD2AP, CD33, CR1, HLA-DRB1, INPP5D, MEF2C, PTK2B
Hematological system development and function	Proliferation	Proliferation of lymphocytes	1.16E−05	APOE, CD2AP, CD33, CR1, HLA-DRB1, INPP5D, MEF2C, PTK2B
Immunological disease	Systemic autoimmune syndrome	Systemic autoimmune syndrome	1.28E−05	APOE, CD33, CLU, CR1, HLA-DRB1, INPP5D, MS4A6A, PTK2B, SORL1^a^
Connective tissue disorders	Juvenile rheumatoid arthritis	Juvenile rheumatoid arthritis	1.50E−05	APOE, CR1, HLA-DRB1, SORL1
Immunological disease	Juvenile rheumatoid arthritis	Juvenile rheumatoid arthritis	1.50E−05	APOE, CR1, HLA-DRB1, SORL1^a^
Inflammatory disease	Juvenile rheumatoid arthritis	Juvenile rheumatoid arthritis	1.50E−05	APOE, CR1, HLA-DRB1, SORL1^a^
Skeletal and muscular disorders	Juvenile rheumatoid arthritis	Juvenile rheumatoid arthritis	1.50E−05	APOE, CR1, HLA-DRB1, SORL1
Cellular function and maintenance	Phagocytosis	Phagocytosis of phagocytes	2.02E−05	ABCA7, BIN1, INPP5D, TREM2
Cell-to-cell signaling and interaction	Phagocytosis	Phagocytosis of phagocytes	2.02E−05	ABCA7, BIN1, INPP5D, TREM2
Inflammatory response	Phagocytosis	Phagocytosis of phagocytes	2.02E−05	ABCA7, BIN1, INPP5D, TREM2^a^
Cellular function and maintenance	Phagocytosis	Phagocytosis of myeloid cells	2.07E−05	ABCA7, BIN1, INPP5D, TREM2
Cell-to-cell signaling and interaction	Phagocytosis	Phagocytosis of myeloid cells	2.07E−05	ABCA7, BIN1, INPP5D, TREM2
Inflammatory response	Phagocytosis	Phagocytosis of myeloid cells	2.07E−05	ABCA7, BIN1, INPP5D, TREM2^a^
Hematological system development and function	Phagocytosis	Phagocytosis of myeloid cells	2.07E−05	ABCA7, BIN1, INPP5D, TREM2
Cellular development	Proliferation	Proliferation of T lymphocytes	2.69E−05	APOE, CD2AP, CD33, CR1, HLA-DRB1, INPP5D, PTK2B
Cellular growth and proliferation	Proliferation	Proliferation of T lymphocytes	2.69E−05	APOE, CD2AP, CD33, CR1,HLA-DRB1, INPP5D, PTK2B
Hematological system development and function	proliferation	proliferation of T lymphocytes	2.69E−05	APOE, CD2AP, CD33, CR1, HLA-DRB1, INPP5D, PTK2B
Cellular function and maintenance	Phagocytosis	Phagocytosis of cells	3.52E−05	ABCA7, BIN1, CR1, INPP5D, TREM2
Cell-to-cell signaling and interaction	Phagocytosis	Phagocytosis of cells	3.52E−05	ABCA7, BIN1, CR1, INPP5D, TREM2
Inflammatory response	Phagocytosis	Phagocytosis of cells	3.52E−05	ABCA7, BIN1, CR1, INPP5D, TREM2^a^
Cell-to-cell signaling and interaction	Immune response	Immune response of macrophages	3.53E−05	ABCA7, BIN1, INPP5D, TREM2
Inflammatory response	Immune response	Immune response of macrophages	3.53E−05	ABCA7 ,BIN1, INPP5D, TREM2^a^
Cellular function and maintenance	Endocytosis	Endocytosis	4.81E−05	APOE, BIN1, CD2AP, PICALM, SORL1
Hematological system development and function	Cell viability	Cell viability of lymphocytes	7.44E−05	CLU, INPP5D, MEF2C, PTK2B
Cell death and survival	Cell viability	Cell viability of lymphocytes	7.44E−05	CLU, INPP5D, MEF2C, PTK2B
Cardiovascular disease	Infarction	Infarction	2.12E−04	APOE, CLU, CR1, MEF2C, TREM2
Organismal development	Abnormal morphology	Abnormal morphology of thoracic cavity	5.37E−04	APOE, BIN1, CD2AP, INPP5D, MEF2C
Cardiovascular system development and function	Abnormal morphology	Abnormal morphology of heart	7.03E−04	APOE,BIN1,CD2AP,MEF2C
Organismal development	Abnormal morphology	Abnormal morphology of heart	7.03E−04	APOE, BIN1, CD2AP, MEF2C
Organ morphology	Abnormal morphology	Abnormal morphology of heart	7.03E−04	APOE, BIN1, CD2AP, MEF2C
Inflammatory response	Cell movement	Cell movement of macrophages	7.55E−04	APOE, CR1, PTK2B, TREM2^a^
Hematological system development and function	Cell movement	Cell movement of macrophages	7.55E−04	APOE, CR1, PTK2B, TREM2
Cellular Movement	Cell movement	Cell movement of macrophages	7.55E−04	APOE, CR1, PTK2B, TREM2
Immune cell trafficking	Cell movement	Cell movement of macrophages	7.55E−04	APOE, CR1, PTK2B, TREM2^a^
Cellular function and maintenance	Function	Function of blood cells	1.08E−03	APOE, INPP5D, PICALM, PTK2B, TREM2
DNA replication, recombination, and repair	Degradation	Degradation of DNA	1.15E−03	APOE, BIN1, CLU, PTK2B
Cell death and survival	Cell viability	Cell viability	1.31E−03	APOE, CD2AP, CD33, CLU, CR1, INPP5D, MEF2C, PTK2B
Organismal development	Abnormal morphology	Abnormal morphology of body cavity	1.55E−03	APOE, BIN1, CD2AP, INPP5D, MEF2C, PICALM
Tissue development	Accumulation	Accumulation of cells	2.16E−03	APOE, CLU, CR1, INPP5D
Hematological system development and function	Cell movement	Cell movement of myeloid cells	2.30E−03	APOE, CR1, INPP5D, PTK2B, TREM2
Cellular movement	Cell movement	Cell movement of myeloid cells	2.30E−03	APOE, CR1, INPP5D, PTK2B, TREM2
Immune cell trafficking	Cell movement	Cell movement of myeloid cells	2.30E−03	APOE, CR1, INPP5D, PTK2B, TREM2^a^
Inflammatory response	Cell movement	Cell movement of phagocytes	2.34E−03	APOE, CR1, INPP5D, PTK2B, TREM2^a^
Hematological system development and function	Cell movement	Cell movement of phagocytes	2.34E−03	APOE, CR1, INPP5D, PTK2B, TREM2
Cellular movement	Cell movement	Cell movement of phagocytes	2.34E−03	APOE, CR1, INPP5D, PTK2B, TREM2
Immune cell trafficking	Cell movement	Cell movement of phagocytes	2.34E−03	APOE, CR1, INPP5D, PTK2B, TREM2^a^
Cellular assembly and organization	Formation	Formation of filaments	2.35E−03	APOE, CLU, EPHA1, PTK2B
Tissue development	Formation	Formation of filaments	2.35E−03	APOE, CLU, EPHA1, PTK2B
Cell-to-cell signaling and interaction	Adhesion	Adhesion of immune cells	2.45E−03	APOE, CLU, CR1, INPP5D
Hematological system development and function	Adhesion	Adhesion of immune cells	2.45E−03	APOE, CLU, CR1, INPP5D
Tissue development	Adhesion	Adhesion of immune cells	2.45E−03	APOE, CLU, CR1, INPP5D
Immune cell trafficking	Adhesion	Adhesion of immune cells	2.45E−03	APOE, CLU, CR1, INPP5D^a^
Cardiovascular disease	Occlusion	Occlusion of artery	2.98E−03	APOE, CLU, FERMT2, HLA-DRB1, PTK2B
Organismal injury and abnormalities	Mammary tumor	Mammary tumor	3.00E−03	APOE, BIN1, CLU, FERMT2, HLA-DRB1, MS4A4A, SLC24A4
Cancer	Mammary tumor	Mammary tumor	3.00E−03	APOE, BIN1, CLU, FERMT2, HLA-DRB1, MS4A4A, SLC24A4^a^
Reproductive system disease	Mammary tumor	Mammary tumor	3.00E−03	APOE, BIN1, CLU, FERMT2, HLA-DRB1, MS4A4A, SLC24A4
Cancer	Endometrioid carcinoma	Endometrioid carcinoma	3.12E−03	APOE, CLU, CR1, EPHA1, FERMT2, NME8, PICALM, RIN3, SORL1^a^
Cell death and survival	Cell death	Cell death of kidney cells	3.16E−03	APOE, CD2AP, CLU, PTK2B
Renal necrosis/cell death	Cell death	Cell death of kidney cells	3.16E−03	APOE, CD2AP, CLU, PTK2B
Cellular development	Maturation	Maturation of cells	3.29E−03	CLU, INPP5D, PTK2B, TREM2
Organismal development	Abnormal morphology	Abnormal morphology of abdomen	3.36E−03	ABCA7, APOE, CD2AP, INPP5D, PICALM
Cancer	Cancer	Cancer	3.63E−03	ABCA7, APOE, BIN1, CASS4, CD33, CLU, CR1, EPHA1, FERMT2, HLA-DRB1, INPP5D, MEF2C, MS4A4A, PICALM, PTK2B, RIN3, SLC24A4, SORL1^a^
Organismal development	Size	Size of body	3.86E−03	APOE, CELF1, INPP5D, PICALM, SLC24A4
Cell-to-cell signaling and interaction	Activation	Activation of leukocytes	3.96E−03	APOE, CR1, HLA-DRB1, INPP5D, TREM2
Inflammatory response	Activation	Activation of leukocytes	3.96E−03	APOE, CR1, HLA-DRB1, INPP5D, TREM2^a^
Hematological system development and function	Activation	Activation of leukocytes	3.96E−03	APOE, CR1, HLA-DRB1, INPP5D, TREM2
Immune cell trafficking	Activation	Activation of leukocytes	3.96E−03	APOE, CR1, HLA-DRB1, INPP5D, TREM2^a^
Cellular movement	Migration	Leukocyte migration	3.99E−03	APOE, CLU, CR1, INPP5D, PTK2B, TREM2
Immune cell trafficking	Migration	Leukocyte migration	3.99E−03	APOE, CLU, CR1, INPP5D, PTK2B, TREM2^a^
Hematological system development and function	Infiltration	Infiltration of leukocytes	4.71E−03	APOE, CR1, INPP5D, PTK2B
Cellular movement	Infiltration	Infiltration of leukocytes	4.71E−03	APOE, CR1, INPP5D, PTK2B
Immune cell trafficking	Infiltration	Infiltration of leukocytes	4.71E−03	APOE, CR1, INPP5D, PTK2B^a^

^a^Groups of molecules related to cancer, immunity/immunological disease or inflammatory responses/inflammatory disease. The analysis produced an extensive list of gene groups significantly associated with an array of categories; however, we have only presented those with at least four molecules for brevity

*ABCA7, BIN1, INPP5D* and *TREM2* are jointly implicated in three forms of phagocytosis, as well as immune response, suggesting they act in tandem to modify these specific aspects of AD. Similarly, *APOE, CR1, INPP5D, PTK2B and TREM2* are jointly responsible for movement of phagocytes and myeloid cells, indicating another group of closely related genes whose activity affects the same cellular functions. Smaller groups of related molecules may better inform about the disease process than individual gene activities such that the whole phenotype attributed to genetic background is greater than the sum of its parts.

The overlap of molecules among different annotations is important as it suggests these genes do not act in isolation, but work in combination so that their actions with respect to disease risk and pathogenesis are dependent upon each other. For example, genetic variation in *SORL1* may mediate an inflammatory mechanism when occurring alongside genetic variation in *PTK2B*, but may be more relevant to endocytosis when occurring with genetic variation in *CD2AP*. As disease mechanisms become clearer, it will be necessary to view disease risk as a function of multiple genetic variants and their interactions.

## Conclusion

Upon review of the literature, it becomes apparent that the genetic mechanisms implicated in AD are incredibly varied and widely distributed across biological functions. The combination of these findings emphasizes the complexity of the disease and suggests multiple therapeutic targets. It will also be important to consider both genetic and environmental factors when attempting to determine major risk factors, as gene effect sizes may be modulated by external factors and vice versa. One particularly curious observation from this review is the number of genes identified by GWAS of LOAD that also have known roles in cancer risk, severity and therapy response. Additionally, incidence rates of cancer seem to be lower among those affected with AD compared to the general population, and the same can be said of cancer survivors with respect to AD incidence [128]. Ganguli [129] comments on this seemingly inverse relationship between cancer and AD. Given our results from the pathway analysis, this supposed relationship warrants a more deliberate focus in future studies to accurately assess its legitimacy.
